# Identification of the *bZIP* gene family and regulation of metabolites under salt stress in *isatis indigotica*

**DOI:** 10.3389/fpls.2022.1011616

**Published:** 2022-10-04

**Authors:** Ming Jiang, Zhen Wang, Weichao Ren, Song Yan, Nannan Xing, Zhanping Zhang, Hui Li, Wei Ma

**Affiliations:** ^1^ Scientific Research Department, Qiqihar Medical University, Qiqihar, China; ^2^ Pharmacy of College, Heilongjiang University of Chinese Medicine, Harbin, China

**Keywords:** *IibZIP* gene family, *isatis indigotica*, salt stress, co-expression, metabolic profiling

## Abstract

The *bZIP* transcription factor family plays important roles in plant growth and development, response to stress, and regulation of secondary metabolite biosynthesis. The identification and molecular function of *bZIP* gene have been deeply studied in the model plant *Arabidopsis thaliana*, but it has not been reported in the medicinal plant *Isatis indigotica*. In this study, 65 *IibZIP* genes were identified in the genome of *I. indigotica*, which were distributed on seven chromosomes, were highly conserved, could be classified into 11 subgroups. Transcriptomic and metabolomic data for leaves of *I. indigotica* exposed to salt stress were analyzed to construct an *IibZIP* gene co-expression network and metabolite correlation network. Seventeen *IibZIP* genes were co-expressed with 79 transcription factors, and GO and KEGG enrichment analysis showed that most of these genes were associated with abiotic stress and hormone responses of plants. 17 *IibZIP* genes regulated 110 metabolites through 92 transcription factor associations. In addition, *IibZIP23*, *IibZIP38* and *IibZIP51* were associated with six metabolites including three alkaloids (quinoline alkaloid stylopine, indole alkaloids tabersonine and indole-3-acetic acid), flavonoid myricetin 3-O-galactoside, and two primary metabolites 2-hydroxy-6-aminopurine, 3-dehydroshikimic acid were strongly correlated. This study provides data for identification of the *IibZIP* gene family and their regulation of metabolites in response to salt stress.

## Introduction

*Isatis indigotica* Fortune is a biennial herb of the Cruciferae family. The species is widely cultivated in China because of its important medicinal and economic value (Nabors et al., 1967). *I. indigotica* has been used as a Chinese herbal medicine for more than 2000 years (Qu et al., 2021). The dried roots (termed Banlangen or *Isatidis Radix*) and dried leaves (termed Daqingye or *Isatidis Folium*) of *I. indigotica* (Qin et al., 2009) both have the effect of clearing heat, detoxification, cooling the blood, eliminating spots, relieving sore throats, and relieving pain (Chang et al., 2012). They are widely used to treat influenza and upper respiratory tract infections in China, and are effective against the severe acute respiratory syndrome coronavirus (SARS-CoV) (Lin et al., 2005) and influenza A virus subtype H1N1 (Wang et al., 2011). In addition, *I. indigotica* contains the plant indigo dye, which is used as an important raw material in the dye industry (Zhou and Zhang, 2013).

In recent years, the chemical constituents of *I. indigotica* have been studied in detail, including alkaloids, glucosinolates, lignans, flavonoids, organic acids, sterols, anthraquinone, coumarins, and other compounds (Gai et al., 2019). Indole alkaloids are common chemical constituents of *I. indigotica*, of which indole-3-carboxaldehyde and isaindigotone have antiviral activity (Molina et al., 2001). It is worth mentioning that the indole ring can also polymerize with phenylpropanoid or lignan to form indole-phenylpropanoid polymers or indole-lignan polymers (Wei-Yang, 2011). Lignan is also an important secondary metabolite of *I. indigotica*, which is the material basis of the antiviral effect of Banlangen (Shi et al., 2012). Lignans have important biological functions both in medicine and in plant defense (Liu et al., 2018). In addition, flavonoids have been isolated from *I. indigotica* in the form of aglycones, such as liquiritigenin, isoliquiritigenin, and neohesperidin (Yi et al., 2003). Modern pharmacological studies have shown that *I. indigotica* has antibacterial, antiviral, antitumor, and anti-inflammatory effects. The market demand for *I. indigotica* continues to grow. Therefore, improvement in the contents of active metabolites will further enhance the effectiveness of *I. indigotica*.

Transcription factors are regulatory factors that control gene expression at the transcriptional level and play crucial roles in plant development and physiology (Sun and Oberley, 1996).The Basic Leucine Zipper (bZIP) transcription factors are among the largest regulatory families of transcription factors and have been identified in almost all eukaryotes, but plants harbor greater diversity of *bZIP* transcription factors than microorganisms and animals (Mijing et al., 2021). For example, 75 *bZIP* transcription factors have been identified in *Arabidopsis thaliana* (Jakoby et al., 2002), 89 in rice (Nijhawan et al., 2008), 47 in birch (Jing et al., 2022), and 84 in pear (Li et al., 2022). The *bZIP* transcription factor is comprised of two parts: one is a highly conserved alkaline region at the N-terminus, which is a DNA recognition domain with sequence-specific DNA-binding ability; the other part is the low-conservative Leucine zipper region at the C-terminus (Cengiz et al., 2014). According to the DNA-binding specificity, bZIP proteins can be classified into four basic types: binding to G-box motifs, binding to C-box motifs, binding to both G-box and C-box motifs, and not forming dimers with DNA (Izawa et al., 1993). By binding to these specific cis-elements, *bZIP* transcription factors form dimers and regulate downstream genes to participate in various processes of plant growth and development.

Abiotic stresses, such as high salinity, drought, and low temperature, have a marked impact on plant growth and development, and may even lead to plant death (Golldack et al., 2014). The bZIP, NAC, AP2/ERF, MYB, and other major plant transcription factor families form a regulatory network in response to abiotic stress, in which the *bZIP* family participates in response to salt, drought, and cold damage, mechanical damage, and osmotic stress (Yu et al., 2022). In addition, many studies on the role of *bZIP* transcription factors in regulating the synthesis of secondary metabolites have been reported. For example, in persimmon, *DkbZIP5* activates *DkMyb4* in response to abscisic acid signaling to increase proanthocyanidin synthesis (Takashi et al., 2012); the *bZIP* family transcription factor *HY5* in *A. thaliana* positively regulates the terpene synthase gene *AtTPS03* and increases terpenoid biosynthesis (Michael et al., 2020); and *RsbZIP011* and *RsbZIP102* in radish are potential participants in the anthocyanin biosynthesis pathway (Lianxue et al., 2019).

High-quality genome sequencing data for *I. indigotica* has been reported (Minghui et al., 2020). However, identification of the *bZIP* gene family in *I. indigotica* has not been studied previously. Considering that the *bZIP* gene family plays important roles in plant growth, development, and stress resistance, the present study aimed to identify the members of the *IibZIP* gene family at the genome-wide level, and to comprehensively analyze the chromosomal location, phylogenetic relationships, gene structure, and *cis*-elements in the promoter region. In addition, through transcriptome–metabolome association analysis, the expression patterns, co-expression network, and metabolite regulation of *IibZIP* genes under exposure to salt stress were studied, which provided valuable information for functional identification of *I. indigotica IibZIP* genes responsive to salinity stress.

## Materials and methods

### Plant materials

*I. indigotica* seeds were obtained from plants cultivated at Daqing City, Heilongjiang Province, and were identified by Ma Dezhi, deputy director of Qiqihar Medical College. The seeds were germinated in an incubator in the laboratory of Qiqihar Medical College. The seedlings were grown under a 14 h/24°C (light) and 10 h/22°C (dark) photoperiod/temperature cycle. Leaves of 6-week-old seedlings were treated with 200 mM NaCl to induce salt stress, and were sampled at 0 h (control group), 24 h, and 48 h. Three plants of uniform growth were selected for each treatment as biological replicates. All samples were immediately frozen in liquid nitrogen and stored at −80°C until use.

### Data sources

The complete genome sequence and annotated file for *I. indigotica* was downloaded from figshare (https://figshare.com/). The *Arabidopsis thaliana* genome assembly (GCA_000005425.2) and annotation file were downloaded from the National Center for Biotechnology Information (NCBI) database (https://www.ncbi.nlm.nih.gov/).

### Identification of *IibZIP* gene family

To identify the members of the *I. indigotica bZIP* gene family, the known bZIP protein sequences of *A. thaliana* were used as the query sequences. The local BLAST program in the TBtools software (Chen et al., 2020) was used for sequence alignment and the E value was set to e^−10^ to search for possible members of the *bZIP* gene family in the *I. indigotica* genome. The obtained candidate sequences were compared with sequences in the NCBI protein database using the BLASTp tool. The candidate sequences were submitted to the NCBI Batch Web CD-Search Tool (https://www.ncbi.nlm.nih.gov/Structure/bwrpsb/bwrpsb.cgi) and Pfam database (http://pfam.xfam.org/) for further identification and screening of the conserved domains. After removing sequences with incomplete domains and redundant sequences, members of the *I. indigotica bZIP* gene family were obtained. The physicochemical properties of the *I. indigotica* bZIP proteins were predicted using the ProtParam tool of the ExPASy website (http://web.expasy.org/protparam/). RNA sequencing (RNA-seq) data from the three *I. indigotica* leaf samples were acquired and uploaded to the NCBI Sequence Read Archive (accession no. SUB11718505) and to BioProject (ID: PRJNA854335).

### Analysis of chromosomal location and duplication of *IibZIP* genes

By analyzing the annotation files of the *I. indigotica* genome, the sequence of the *I. indigotica bZIP* genes was mapped to individual chromosomes. The chromosomal location of each *bZIP* gene was determined and a corresponding chromosomal physical location map was drawn. The genes were named in accordance with the order of their chromosomal location. Duplication events for the *bZIP* genes in *I. indigotica* were predicted using MCScanX (Wang et al., 2012). The Dual Systeny Plot function of the TBtools software was used to visually analyze the genomic collinearity between *I. indigotica* and various monocotyledons and dicotyledons, and to highlight the synteny relationships of the *bZiP* genes.

### Gene structure and phylogenetic analysis of *IibZIP*

The *bZIP* motifs were analyzed using the MEME Suite tools (https://meme-suite.org/meme/tools/meme). The parameters were set to ZOOPS for site distribution and 10 for the number of motifs. The upstream 2000 bp sequence of each *IibZIP* gene was defined as the promoter region, and the promoter sequence was extracted and submitted to the PlantCARE website (http://bioinformatics.psb.ugent.be/webtools/plantcare/html/). The number of cis-elements were counted. The TBtools software was used to analyze and visualize the conserved domain, exon, intron, motif, and *cis*-element composition of the *bZIP* genes. The neighbor-joining method implemented in MEGA-X software (Sudhir et al., 2018) was used to construct a phylogenetic tree for the bZIP proteins of *I. indigotica* and *A. thaliana*. ClustalW was used to generate a multiple alignment of the amino acid sequences. The phylogenetic analysis included 1000 bootstrap replicates and the default values were used for other settings.

### Transcriptomic analysis and qRT-PCR verification of *IibZIP*

The fragments per kilobase of exon model per million mapped fragments (FPKM) value of each *bZIP* gene was extracted from the RNA-seq data for *I. indigotica* leaves under different durations of salt stress. A heatmap of *bZIP* gene expression was constructed using the TBtools software to analyze expression patterns. The FPKM values were processed using row-scaling transformation.

The RNA-seq data were verified by conducting a quantitative real-time PCR (qRT-PCR) analysis. Total RNA was extracted from the *I. indigotica* leaf samples treated with salt stress using the Plant Total RNA Kit (SIMGEN, Hangzhou, China). The RNA was reverse-transcribed into cDNA using the SureScript™ First-Strand cDNA Synthesis Kit (iGeneBio, Guangzhou, China). The qRT-PCR experiment was performed using the cDNA as the template using the BlazeTaq™ SYBR^®^ Green qPCR Mix 2.0 Kit (iGeneBio) in accordance with the manufacturer’s protocol. The IiMUB gene (GARR01001157.1) was used as the internal reference gene (Qu et al., 2019). The relative expression level of each gene was calculated using the 2^−△△Ct^ algorithm. Three technical replicates were analyzed in each experiment. The qRT-PCR primers were designed using Primer Premier (version 5.0) software. All primers used in this study were synthesized by Rui Biotech Biotechnology (Harbin) Co., Ltd.

### Untargeted metabolic profiling

Nine samples of *I. indigotica* leaves (one group from non-stressed plants, two groups from the salt stress treatment for 24 h and 48 h, and three biological replicates for each group) were chemically extracted for metabolic analysis. The samples were incubated on ice for 5 min and then centrifuged at 15,000 g at 4°C for 20 min. Some supernatants were diluted to the final concentration containing 53% methanol with LC-MS grade water. The samples were then transferred to a fresh Eppendorf tube and centrifuged at 15,000 g at 4°C for 20 min. The supernatant was injected into the liquid chromatography–tandem mass spectrometry (LC-MS/MS) system for analysis (Want et al., 2013).

The ultra-high-performance LC-MS/MS analyses were performed using a Vanquish UHPLC system (Thermo Fisher, Bremen, Germany) coupled with an Orbitrap Q Exactive™ HF mass spectrometer (Thermo Fisher) by Novogene Co., Ltd. (Beijing, China). Samples were injected onto a Hypersil GOLD column (100 2.1 mm, 1.9 μm) using a 17-min linear gradient at a flow rate of 0.2 mL/min. The eluents for the positive polarity mode were eluent A (0.1% formic acid in water) and eluent B (methanol). The eluents for the negative polarity mode were eluent A (5 mM ammonium acetate, pH 9.0) and eluent B (methanol). The solvent gradient was set as follows: 2% B, 1.5 min; 2%–100% B, 3 min; 100% B, 10 min; 100%–2% B, 10.1 min; and 2% B, 12 min. The Q Exactive™ HF mass spectrometer was operated in positive/negative polarity mode with spray voltage 3.5 kV, capillary temperature 320°C, sheath gas flow rate 35 psi, auxiliary gas flow rate 10 L/min, S-lens RF level 60, and auxiliary gas heater temperature 350°C.

The metabolites were annotated using the Kyoto Encyclopedia of Genes and Genomes (KEGG) pathway database

(https://www.genome.jp/kegg/pathway.html), Human Metabolome Database (https://hmdb.ca/metabolites), and LIPID Maps database (http://www.lipidmaps.org/).

### Statistical analysis

Transcription factor prediction in the entire genome of *I. indigotica* was performed using the PlantTFDB database (http://planttfdb.gao-lab.org/prediction.php) (Tian et al., 2019). Conserved domain annotation was performed using the Pfam database (http://pfam.xfam.org/) (Sara et al., 2018). Annotation with gene ontology (GO) terms and KEGG pathways was performed using eggNOG-mapper (http://eggnog-mapper.embl.de/) (Cantalapiedra et al., 2021).

We applied the R package ‘DESeq2’ to filter the differentially expressed genes (DEGs) in accordance with the FPKM values (Love et al., 2014). Significant DEGs were selected with the following criteria: |log2(fold change)| ≥ 1 and Padj ≤ 0.05. The R package ‘clusterProfiler’ was used for GO and KEGG enrichment analysis (Wu et al., 2021).

Transcriptome and metabolome correlations were calculated using Python scripts (Zhang et al., 2017). Correlation coefficients were calculated using Pearson correlation analysis. Co-expression networks of genes and metabolites were visualized using Cytoscape (version 3.7.1) software (Shannon and P., 2003). Metabolite relative content was plotted using GraphPad Prism (version 6.0) software and the significance of differences were analyzed using Student’s t-test.

## Results

### Identification and characterization of *bZIP* gene family members in *I. indigotica*

To identify the *bZIP* gene family members in the *I. indigotica* genome, we used the BLASTp tool and a Hidden Markov Model to scan the entire genome, and then verified the sequence of the conserved domain with the CD-Search tool and the Pfam database. In total, 65 *IibZIP* genes were confirmed in the *I. indigotica* genome (**Supplementary Table 1**). In accordance with their order of distribution on the chromosomes, the genes were named *IibZIP1* to *IibZIP65*. The number of amino acids encoded by the individual *IibZIP* gene sequences ranged from 114 to 723, the molecular weight ranged from 11,124.45 to 74,247.06 Da, and the isoelectric point ranged from 4.75 to 10.1. All IibZIP proteins were predicted to be hydrophilic (**Supplementary Table 1**).

**Figure 1:**
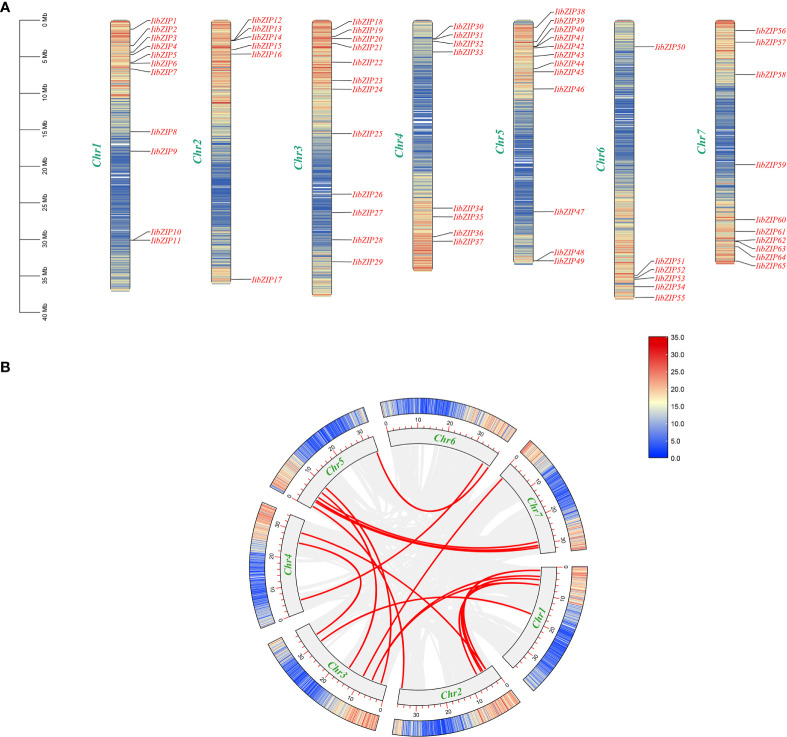
Chromosomal distribution of *IibZIP* genes and predicted gene replication events in the (*I*) *indigotica* genome. **(A)** Location of *IibZIP* genes on *I indigotica* chromosomes. **(B)** Gene duplication events in the genome. A red line represents collinearity of the *IibZIP* gene, and a gray line represents all collinear pairs in the (*I*) *indigotica* genome.

The 65 *IibZIP* genes were distributed on seven chromosomes and contigs in *I. indigotica*, but no obvious distribution pattern was observed (**Figure 1A**). Chromosome 3 carried the highest number of *IibZIP* genes (16), whereas chromosome 6 had the fewest *IibZIP* genes (7). No genes were distributed on contigs.

To facilitate investigation of *I. indigotica* gene replication events and to explore the expansion of the *IibZIP* gene family, all contigs of *I. indigotica* were deleted. The *IibZIP* gene duplication events were analyzed using MCScanX (**Supplementary Table 2**) (**Figure 1B**). Sixteen pairs of gene segment duplication events were identified on seven chromosomes. These *IibZIP* gene segment duplication events may have played an important role in the evolution of *I. indigotica.*

### Phylogenetic analysis of IibZIP proteins

**Figure 2:**
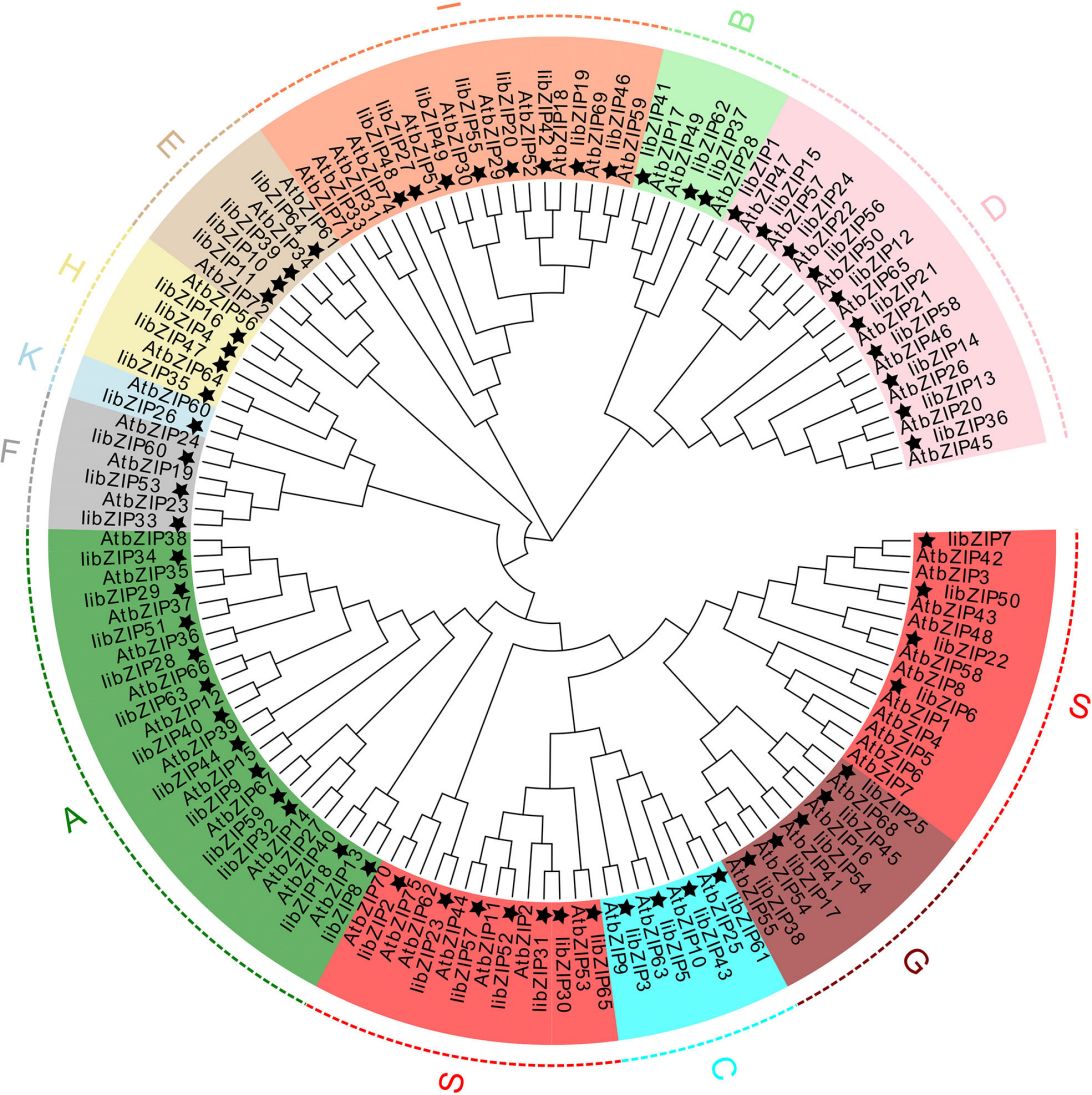
Phylogenetic tree for bZIP proteins of *I. indigotica* and *A.thaliana* thaliana. The topology was assessed with a bootstrap analysis with 1000 replicates. A star indicates the IibZIP proteins, and different colors and letters represent different subgroups.

To evaluate the evolutionary relationships of *I. indigotica* and *A. thaliana* bZIP proteins, a neighbor-joining phylogenetic tree was constructed (**Figure 2**). All IibZIP and AtbZIP proteins were clustered (**Supplementary Table 3**). Consistent with the classification of AtbZIP proteins, the IibZIP proteins were divided into 11 subgroups, comprising A (12), B (3), C (4), D (10), E (4), F (3), G (5), H (4), I (8), K (1), and S (11). Interestingly, the IibZIP proteins in the S subgroup were not tightly clustered, unlike this subgroup in *A.thaliana*.

### Gene structure and *cis*-element analysis of *IibZIP* genes

**Figure 3:**
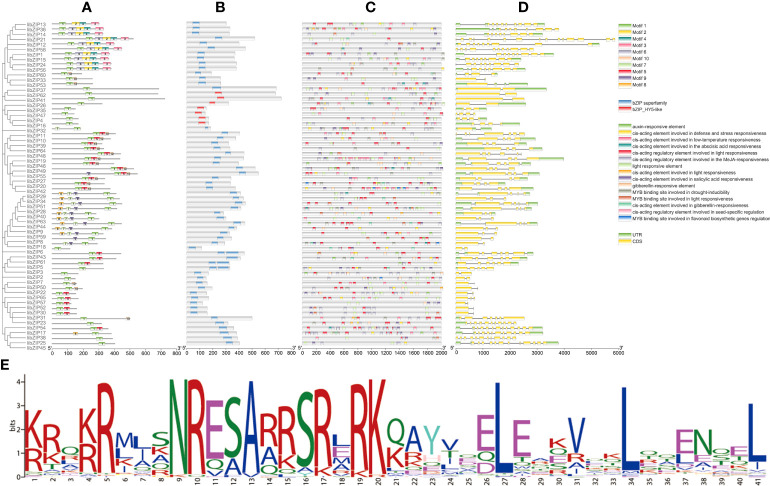
Gene structure and *cis*-element prediction of *IibZIP* proteins. **(A)** Motif analysis of *IibZIP* proteins. Different numbers represent different motifs. **(B)** Conserved domain and location information for each *IibZIP* gene. **(C)** *Cis*-elements in the promoter region of *IibZIP* genes. Different colored rectangles represent different *cis*-element components. **(D)** Structure of *IibZIP* genes. A green rectangle indicates an untranslated region; a yellow rectangle indicates the coding sequence region; lines indicate introns. **(E)** Amino acid sequence information for motif 1. The x-axis of **(A, B)** is the number of amino acids. The X-axis of **(B, D)** is the number of nucleotides.

In plants, *bZIP* genes contain two functional domains: a basic region and an adjacent leucine zipper region. The basic region is highly conserved in eukaryotes and is the DNA-binding domain, which generally consists of 18 amino acid residues and has the same amino acid sequence model (N–X_7_–R/K–X_9_). The leucine zipper region is composed of the repeated amino acid sequence L–X_6_–L–X_6_–L, which varies greatly. Leucine is located at the seventh amino acid of the heptapeptide sequence and can be replaced by isoleucine, valine, phenylalanine, or methionine. We analyzed all IibZIP protein sequences (**Figure 3**). Predictions of motifs (**Figure 3A**) and conserved domains (**Figure 3B**) revealed that each subgroup had the same motifs, which may be associated with evolutionary conservation. All *IibZIP* proteins had motif 1 regions, which were composed of the basic region and the leucine zipper of the *bZIP* transcription factor; these regions are crucial for formation of the *bZIP* dimer and for binding to DNA to exert its regulatory role. The *bZIP* superfamily domain was detected in all *IibZIP* genes, and seven genes contained the bZIP-HY5-like domain.

A *cis*-element is a non-coding sequence in a gene promoter region that regulates the transcription of related genes. In this study, the upstream 2000 bp of each *IibZIP* gene was extracted as the promoter region for analysis (**Figure 3C**). The *cis*-acting elements in the promoter region of the *IibZIP* genes were diverse in type and number (**Supplementary Table 4**). Some *cis*-elements were associated with hormone regulation, including auxin, gibberellin, abscisic acid, and methyl jasmonate responsive elements. Certain *cis*-elements were involved in abiotic stress response, including elements involved in defense and stress response and low-temperature response elements. Plant growth- and development-related elements included light response elements, salicylic acid response elements, and seed-specific regulatory response elements. Further analysis revealed that the *IibZIP* genes contained a binding site for a *MYB* gene involved in light response, drought response, and flavonoid synthesis regulation. It is possible that the IibZIP protein interacts with the *IiMYB* gene and forms a regulatory network.

To better characterize the *IibZIP* gene structure, we investigated the distribution of the coding sequence, untranslated regions, and introns (**Figure 3D**). The number of introns ranged from 1 to 14, and the number of introns in the same subgroup of *IibZIP* genes was similar. The difference in number of introns and exons of the *IibZIP* genes reflected the structural diversity of the *bZIP* gene family members of *I. indigotica*.

### Expression pattern and qRT-PCR validation of *IibZIP* genes under abiotic stress

**Figure 4:**
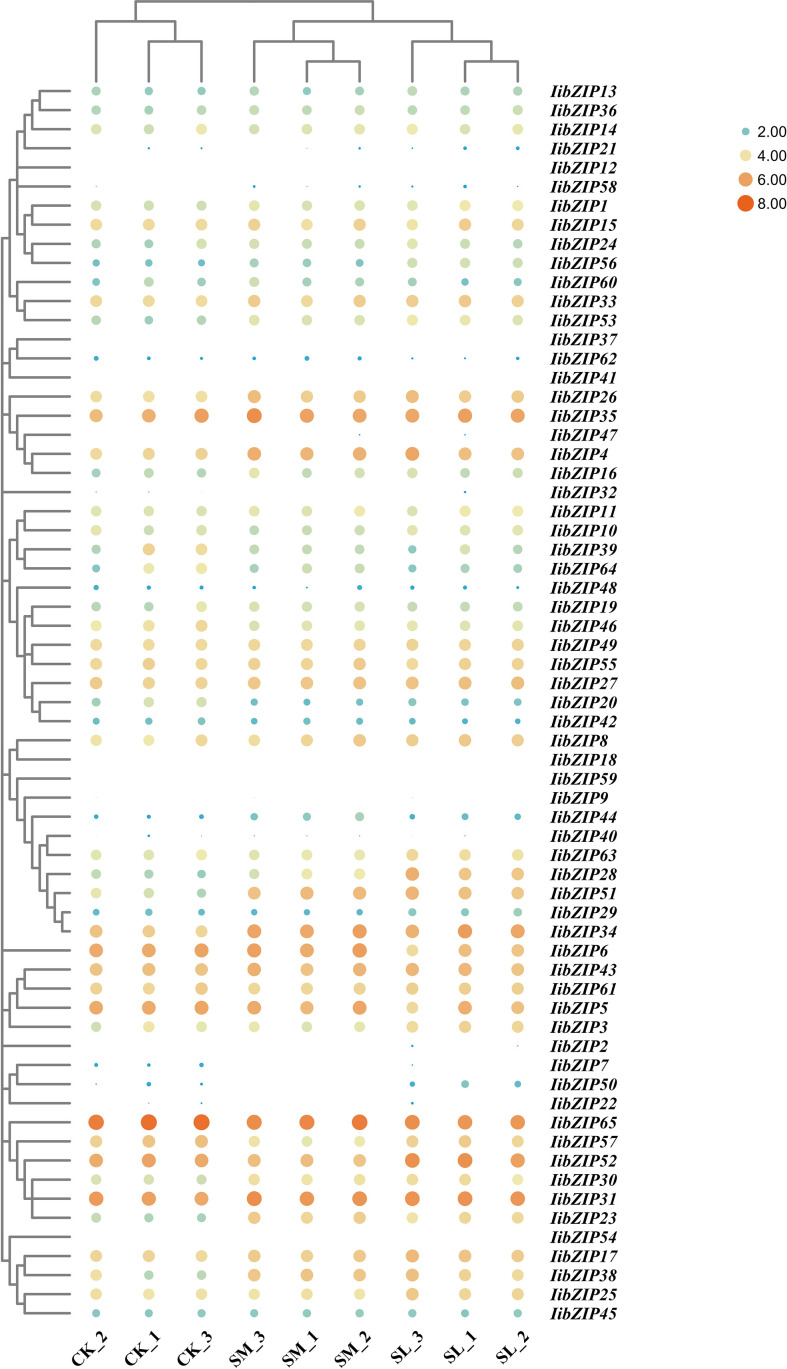
Heatmap of *IibZIP* gene expression in *I. indigotica* leaves under salt stress for different durations. The FPKM values were processed using row-scaling transformation. The dot size indicates the degree of gene expression. Different colors indicate the degree of gene expression: orange indicates higher expression and blue lower expression. CK, non-stress environment; SM, salt stress treatment for 24 h; SL, salt stress treatment for 48 h. Each group comprised three biological replicates.

Overall changes in multiple samples and genes can be visualized through heatmaps. To reveal the expression pattern of *IibZIP* genes in the leaf under salt stress for different durations, we extracted the FPKM values of the *IibZIP* genes from the RNA-seq data and generated heatmaps to compare gene expression patterns in the non-stress environment (CK), under 24 h salt stress (SM), and 48 h salt stress (SL) (**Supplementary Table 5**) (**Figure 4**). The expression of 28 *IibZIP* genes was increased under salt stress, nine *IibZIP* genes showed decreased gene expression, and expression of 13 *IibZIP* genes was not detected (FPKM < 0.5). After 24 h salt stress, the expression of *IibZIP44* changed the most (increased by 5.2 times). After 48 h salt stress, the expression of I*ibZIP50* changed the most (increased by 5.1 times). These positively regulated *IibZIP* genes under salt stress may help *I. indigotica* to tolerate abiotic stress and promote plant growth and development.

**Figure 5:**
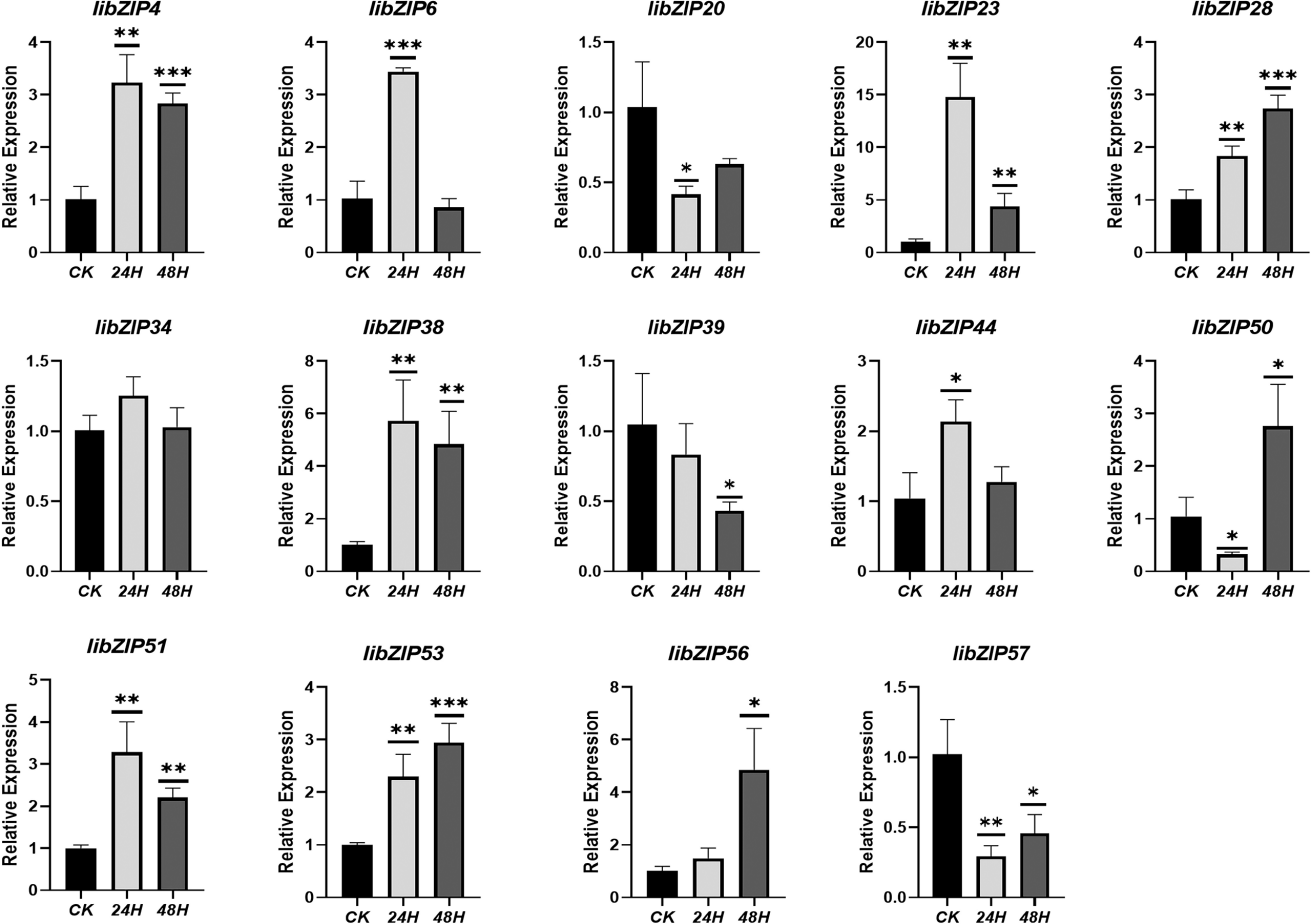
Relative expression levels of 14 differentially expressed *IibZIP* genes in *I. indigotica* leaves under salt stress for different durations detected by qRT-PCR. The X-axis indicates salt stress duration, the Y-axis indicates relative gene expression level. CK, non-stress environment. Error bars indicate the standard deviation of three repetitions. Asterisks denote a statistically significant difference (*p < 0.05, **p < 0.01, ***p < 0.001; Student’s t-test).

To characterize the specific expression patterns of the *IibZIP* genes under salt stress, their expression under salt stress for different durations was compared in RNA-seq data (**Supplementary Table 6**). A total of 14 *IibZIP* genes were differentially expressed (p < 0.05). Compared with the non-stress environment, seven DEGs were detected under both 24 h and 48 h salt stress. Five DEGs were specific to the 24 h salt treatment, namely *IibZIP6*, *IibZIP28*, *IibZIP50*, *IibZIP53*, and *IibZIP56*. Two DEGs were specific to the 48 h salt stress treatment, namely *IibZIP4* and *IibZIP57*. The relative transcript abundances of the 14 DEGs were validated using qRT-PCR to determine the reliability of the RNA-seq data (**Figure 5**). The expression trends of all DEGs in most qRT-PCR analyses were consistent with the RNA-seq data, but there were also deviations, such as libZIP4 was significantly expressed under both treatments, libZIP34 was not significantly expressed under both treatments, libZIP56 Gene expression is the opposite of RNA-seq data. It is worth noting that the FPKM value of *IibZIP50* under 24 h salt stress was zero. Although the qRT-PCR result was clear, the expression level was much lower than that detected in the non-stress environment and 48h salt stress treatment, which was consistent with the expression trend for *IibZIP50* in the RNA-seq data.

### Co-expression network of salt-mediated transcriptional reprogramming and enrichment analysis

**Figure 6:**
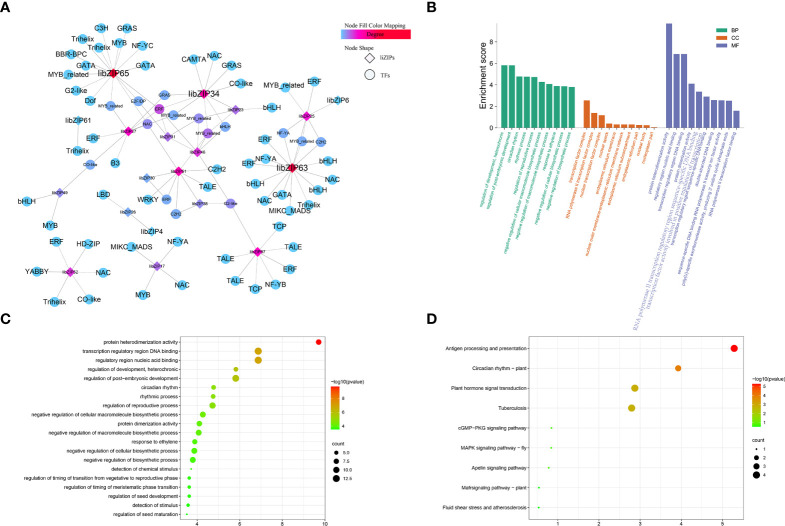
*I. indigotica IibZIP* gene co-expression network, and GO term and KEGG pathway enrichment analysis. **(A)** Co-expression network of *IibZIP* genes constructed based on the transcriptome of *I. indigotica* leaves under salt stress. Diamonds indicate the *IibZIP* genes and circles indicate other transcription factors. The color shade indicates the number of relevant genes. **(B)** GO term annotations of *IibZIP* genes and co-expressed transcription factors. BP, biological processes; CC, cellular components; MF, molecular functions. **(C)** GO term enrichment analysis of *IibZIP* genes and co-expressed transcription factors. **(D)** KEGG pathway enrichment analysis of *IibZIP* genes and co-expressed transcription factors.

When plants are exposed to abiotic stress, the *bZIP* genes usually form a regulatory network with a variety of other transcription factors to help plants to tolerate the stress. To explore the transcriptional regulatory network of *IibZIP* genes under exposure to salt stress, the transcription factors detected in the transcriptome of *I. indigotic*a leaves under salt stress were identified. A total of 1766 transcription factors were detected, which were classified into 58 transcription factor families (**Figure 6A**), among which the five most abundant families were bHLH (n = 161 genes), ERF (n = 123), MYB (n = 122), NAC (n = 105), and MYB-related (n = 84) (**Supplementary Table 7**). Using a Python script, the expression levels of all transcription factor genes under salt stress were extracted and co-expression analysis was performed with the *IibZIP* genes (**Figure 6A**). A total of 17 *IibZIP* genes were co-expressed with 79 transcription factors (r > 0.9). The five transcription factor families most frequently co-expressed were MYB-related (14), ERF (13), NAC (8), bHLH (7), and Trihelix (5). These transcription factor families have been confirmed to participate in abiotic stress responses of plants in previous studies. Therefore, when *I. indigotica* is subjected to salinity stress, the *IibZIP* genes may form a regulatory network with other transcription factors to help the plant to adapt and to prevent stress-related damage.

To explore the functions of these transcription factors, GO annotation analysis was performed on the 17 *IibZIP* genes and 79 transcription factors in the co-expression network (**Figure 6B**). The transcription factors were mainly involved in the regulation of plant development, circadian rhythm, negative regulation of cellular macromolecule biosynthesis, and response to ethylene in the biological process category. In the cellular component category, the transcription factors were mainly annotated with transcription factor complex, RNA polymerase II transcription factor complex, nuclear transcription factor complex, and nuclear speck. With regard to molecular functions, the transcription factors were mainly annotated with protein heterodimerization activities, regulatory region nucleic acid binding, transcription regulatory region DNA binding, and protein dimerization activity. These molecular functions are mainly involved in transcriptional regulation and protein expression activation.

In addition, the genes in the co-expression network were subjected to GO term and KEGG pathway enrichment analyses (**Supplementary Table 8**). The GO enrichment analysis revealed that the main functions enriched among the *IibZIP* genes and co-expressed transcription factors were protein heterodimerization activity, regulatory region nucleic acid binding, transcription regulatory region DNA binding, regulation of development, heterochronic, regulation of post-embryonic development, circadian rhythm, and numerous other processes (**Figure 6C**). The KEGG pathway enrichment analysis indicated that the co-expressed genes were mainly enriched in plant circadian rhythm regulation, plant hormone signal transduction, MAPK signal pathway, and other pathways (**Figure 6D**). These results indicated that the *IibZIP* genes participated in a diverse variety of biological processes.

### *IibZIP* genes participate in metabolic regulation under salt stress

**Figure 7:**
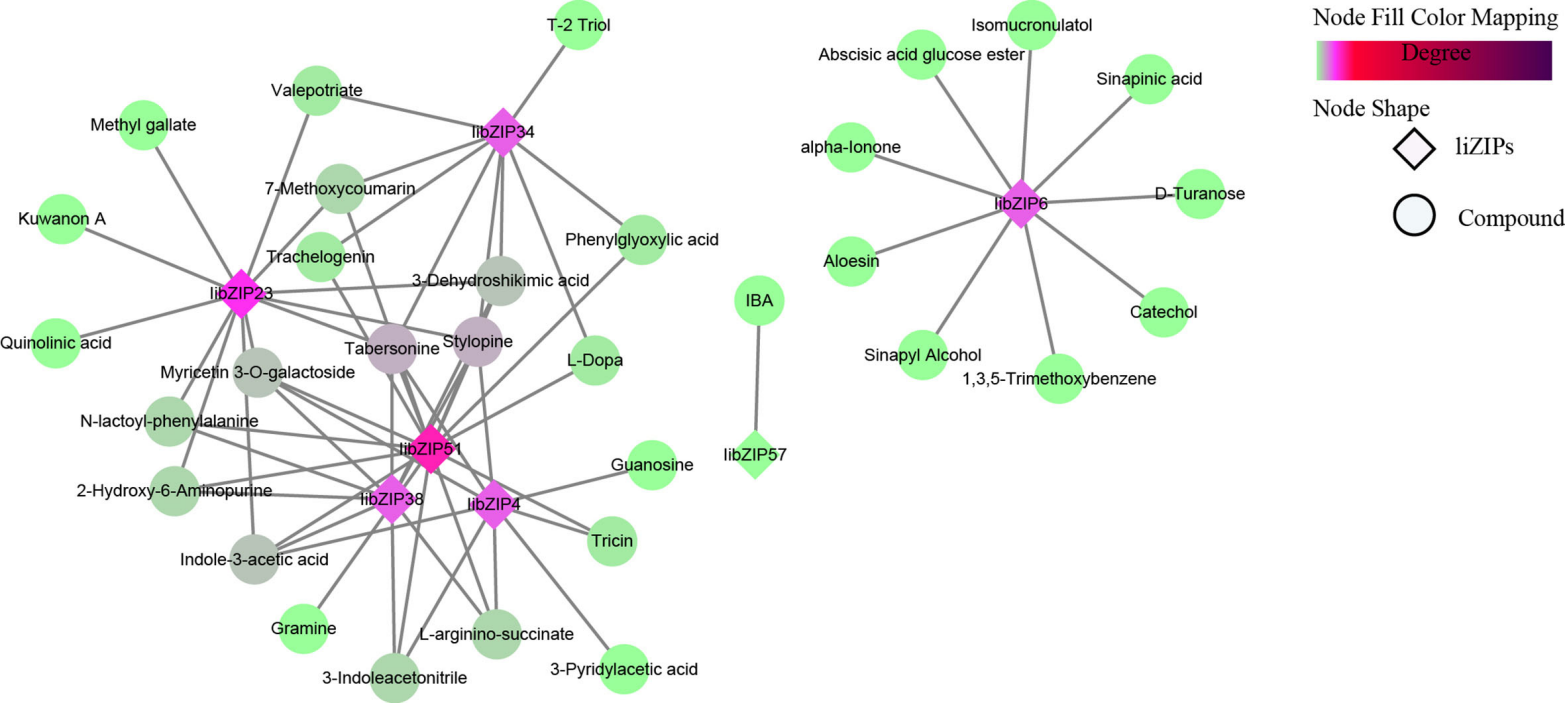
Pearson correlation network revealing the regulation by *IibZIP* genes of metabolite synthesis in leaves of *I. indigotica* under salt stress. Diamonds indicate *IibZIP* genes and circles indicate metabolites. The color shade indicates the number of genes associated with metabolites.

Tto study the involvement of *IibZIP* genes in plant metabolic regulation under salt stress, the leaf transcriptomic and metabolomic data under salt stress were integrated to conduct a correlation network analysis between differentially expressed *IibZIP* genes in the transcriptome and up-regulated metabolites in the metabolome (**Figure 7**). In total, 39 nodes and 64 network pairs (r > 0.7), including seven *IibZIP* genes and 32 metabolites, were positively correlated (**Supplementary Table 9**). Seven *IibZIP* gene members were significantly correlated with the up-regulated metabolites, namely *IibZIP51* (degree = 15), *IibZIP23* (degree = 12), *IibZIP4* (degree = 9), *IibZIP34* (degree = 9), *IibZIP38* (degree = 9), *IibZIP6* (degree = 9), and *IibZIP57* (degree = 1). The six metabolites with the highest correlation coefficients with *IibZIP* genes included three alkaloids, comprising the quinoline alkaloid stylopine (count = 5) and the indole alkaloids tabersonine (count = 5) and indole-3-acetic acid (count = 4). The latter compound is an important natural plant growth hormone. In addition, the flavonoid myricetin 3-O-galactoside (count = 4) and the primary metabolites 2-hydroxy-6-aminopurine (count = 3) and 3-dehydroshikimic acid (count = 4) were strongly correlated with the *IibZIP* genes (**Table 1**).

**Table 1:** Correlation analysis of differentially expressed *IibZIP* genes and differentially accumulated metabolites in leaves of *I. indigotica* under salt stress.

Gene_id	Compound	Molecular formula	Degree	Correlation
*IibZIP51*	Tabersonine	C_21_H_24_N_2_O_2_	5	0.787381**
*IibZIP23*	Tabersonine	C_21_H_24_N_2_O_2_	5	0.893247**
*IibZIP4*	Tabersonine	C_21_H_24_N_2_O_2_	5	0.738214**
*IibZIP34*	Tabersonine	C_21_H_24_N_2_O_2_	5	0.791785**
*IibZIP38*	Tabersonine	C_21_H_24_N_2_O_2_	5	0.812325**
*IibZIP51*	Stylopine	C_19_H_17_NO_4_	5	0.837279**
*IibZIP23*	Stylopine	C_19_H_17_NO_4_	5	0.733452**
*IibZIP4*	Stylopine	C_19_H_17_NO_4_	5	0.738612**
*IibZIP34*	Stylopine	C_19_H_17_NO_4_	5	0.707277**
*IibZIP38*	Stylopine	C_19_H_17_NO_4_	5	0.824055**
*IibZIP51*	Myricetin3-O-galactoside	C_21_H_20_O_13_	4	0.820737**
*IibZIP23*	Myricetin3-O-galactoside	C_21_H_20_O_13_	4	0.805632**
*IibZIP4*	Myricetin3-O-galactoside	C_21_H_20_O_13_	4	0.845623**
*IibZIP38*	Myricetin3-O-galactoside	C_21_H_20_O_13_	4	0.931146**
*IibZIP51*	Indole-3-acetic acid	C_10_H_9_NO_2_	4	0.789529**
*IibZIP23*	Indole-3-acetic acid	C_10_H_9_NO_2_	4	0.732771**
*IibZIP4*	Indole-3-acetic acid	C_10_H_9_NO_2_	4	0.887589**
*IibZIP38*	Indole-3-acetic acid	C_10_H_9_NO_2_	4	0.707793**
*IibZIP51*	3-Dehydroshikimic acid	C_7_H_8_O_5_	4	0.914323**
*IibZIP23*	3-Dehydroshikimic acid	C_7_H_8_O_5_	4	0.777244**
*IibZIP34*	3-Dehydroshikimic acid	C_7_H_8_O_5_	4	0.819383**
*IibZIP38*	3-Dehydroshikimic acid	C_7_H_8_O_5_	4	0.837108**
*IibZIP23*	2-Hydroxy-6-Aminopurine	C_5_H_5_N_5_O	3	0.701065**
*IibZIP38*	2-Hydroxy-6-Aminopurine	C_5_H_5_N_5_O	3	0.835132**
*IibZIP51*	2-Hydroxy-6-Aminopurine	C_5_H_5_N_5_O	3	0.771324**

Correlation>0.7: positive regulation. ** means p-value < 0.01.

**Figure 8:**
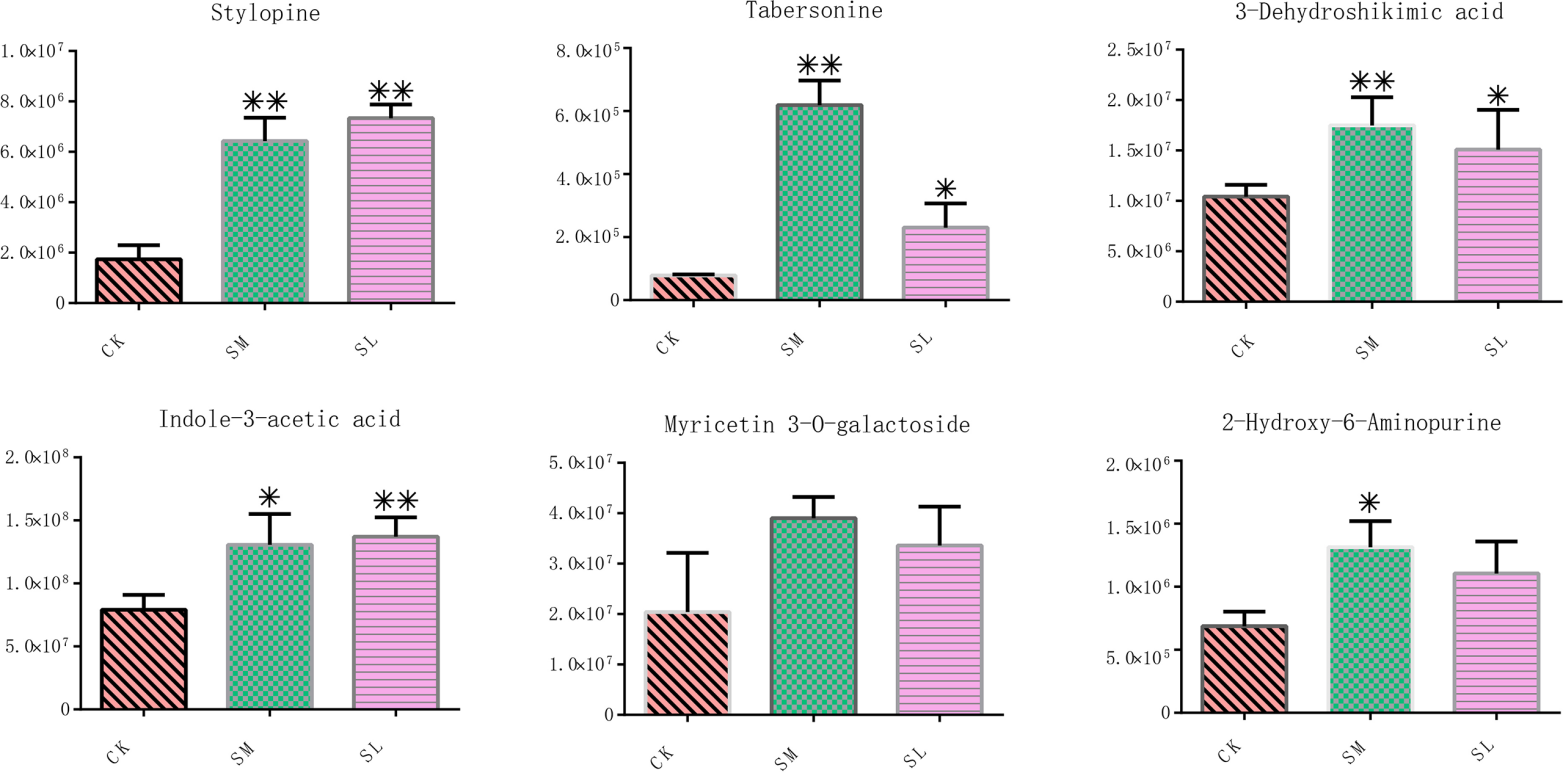
Relative contents of six metabolites with the highest correlation coefficients with *IibZIP* genes in leaves of *I. indigotica* after salt stress treatment. Error bars denote the standard deviation of three replicates (*p < 0.05, **p < 0.01).

The relative contents of these six metabolites were characterized. Accumulation of these metabolites was greatly increased under salt stress compared with the CK group, among which the contents of tabersonine, myricetin 3-O-galactoside, 3-dehydroshikimic acid, and 2-hydroxy-6-aminopurine were highest in the SM group, and the contents of stylopine and indole-3-acetic acid were highest in the SL group (**Figure 8**). Student’s t-tests revealed that, except for myricetin 3-O-galactoside and 2-hydroxy-6-aminopurine, the contents of the other four metabolites in the SM and SL groups were significantly changed compared with those in the CK.

**Figure 9:**
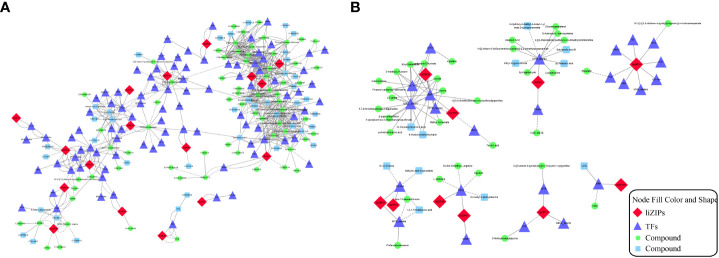
Metabolite association diagram of IibZIP genes and other transcription factors. **(A)** Association diagram of all differentially expressed bZIP genes, transcription factors and metabolites. **(B)** Association diagram of metabolites with the strongest associations with 10 bZIP genes. Different shapes and colors represent different substances. Solid lines indicate positive correlations and dashed lines indicate negative correlations.

To exerts its regulatory function, a *bZIP* gene often forms a regulatory network with different transcription factors. Based on the correlation analysis of *bZIP* genes with metabolites, a combined analysis of the transcriptomic and metabolomic data was conducted to explore the relationships among *bZIP* genes, other transcription factors, and metabolites in metabolic regulation (**Figure 9**). In the constructed network of 746 edges, a total of 219 nodes were connected, with 547 pairs (r > 0.9) for the positively correlated network and 199 pairs (r ≤ 0.9) for the negatively correlated network (**Figure 9A**). The degree of association between each node was expressed as the Pearson correlation coefficient. Among the 219 nodes, 17 *IibZIP* genes were associated with 110 metabolites through 92 transcription factors (**Supplementary Table 10**).

**Figure 10:**
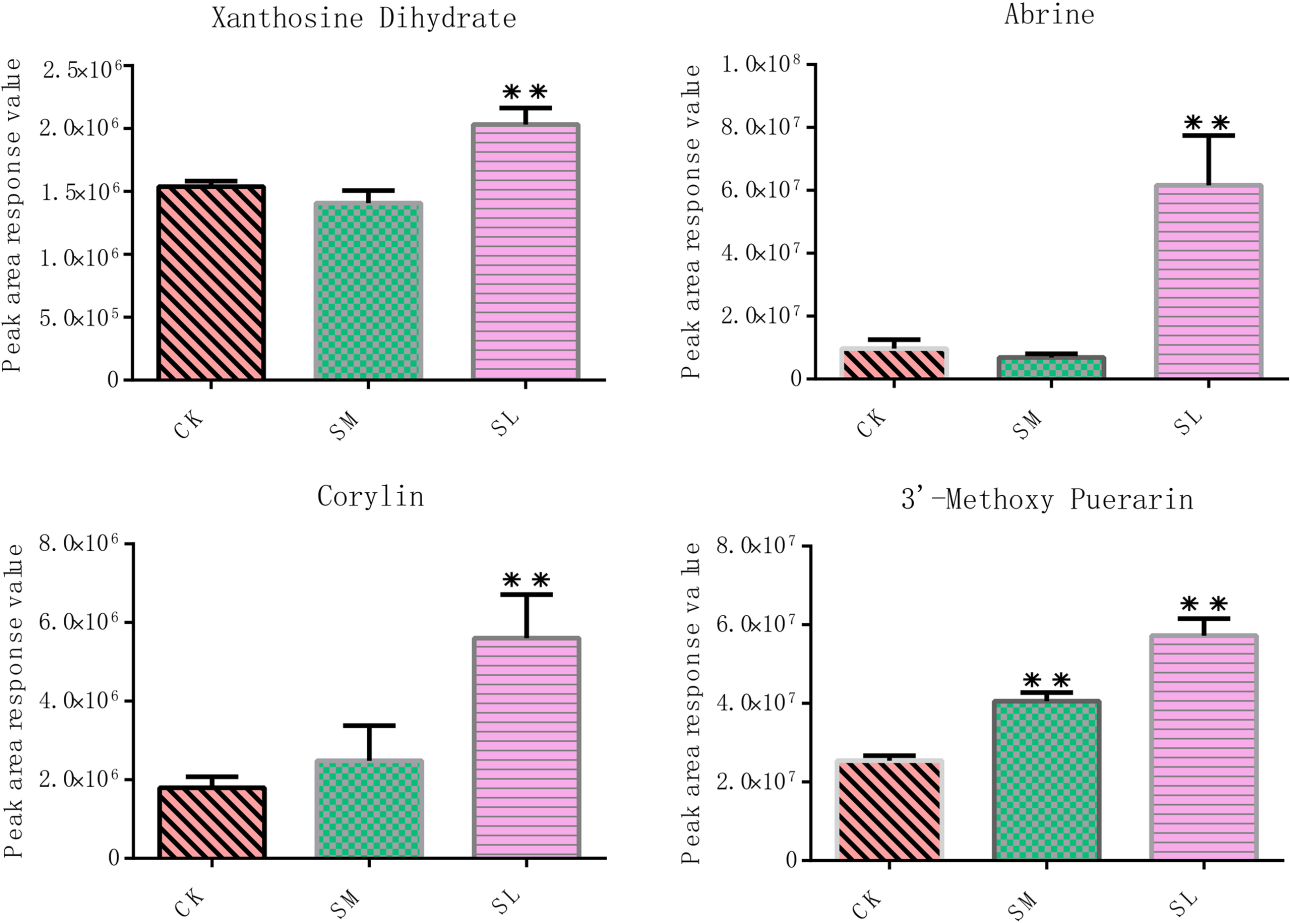
Relative contents of four metabolites most strongly correlated with the *IibZIP* gene regulatory network in leaves of *I. indigotica* after salt stress treatment. Error bars denote the standard deviation of three replicates (*p < 0.05, **p < 0.01).

By integrating the metabolite findings with the highest degree in the network, we constructed 106 network pairs of 145 nodes (**Figure 9A**). Ten *IibZIP* genes, 24 transcription factors, and 44 metabolites were strongly associated (r > 0.9). There were 81 positive regulatory associations and 25 negative regulatory associations (**Figure 9B**). The four *IibZIP* genes with the strongest associations were *IibZIP3*, *IibZIP25*, *IibZIP34*, and *IibZIP63* (**Table 2**). The transcription factors most strongly associated with *IibZIP* genes were MYB-related (count = 4), ERF (count = 3), MIKC_MADS (count = 2), and CO-like (count = 2). The four metabolites with the strongest genetic associations were two flavonoids, corylin (C_20_H_16_O_4_) and 3′-methoxy puerarin (C_22_H_22_O_10_), the indole alkaloid abrine (C_12_H_14_N_2_O_2_), and the primary metabolite xanthosine dihydrate (C_10_H_12_N_4_O_6_). The relative contents of these four compounds were increased under salt stress and the effect of salt stress for a longer period was more strongly significant (**Figure 10**).

**Table 2:** Correlation analysis of the four compounds most strongly associated with the *IibZIP* gene regulatory network in leaves of *I. indigotica* under salt stress.

Gene_id	TFS	Compound	Molecular formula	Degree	Correlation
*libzip25*	Iin08374(MYB_related)	Xanthosine Dihydrate	C10H12N4O6	4	-0.96422**
*libzip25*	Iin10919(NF-YA)	Xanthosine Dihydrate	C10H12N4O6	4	0.964485**
*libzip25*	Iin21617(ERF)	Xanthosine Dihydrate	C10H12N4O6	4	0.979377**
*libzip25*	Iin22265(MIKC_MADS)	Xanthosine Dihydrate	C10H12N4O6	4	0.971282**
*libzip25*	Iin22457(GATA)	Xanthosine Dihydrate	C10H12N4O6	4	0.955758**
*libzip25/libzip63*	Iin08374(MYB_related)	Abrine	C12H14N2O2	4	-0.96626**
*libzip25*	Iin10919(NF-YA)	Abrine	C12H14N2O2	4	0.975134**
*libzip25/libzip63*	Iin21617(ERF)	Abrine	C12H14N2O2	4	0.981377**
*libzip25*	Iin22265(MIKC_MADS)	Abrine	C12H14N2O2	4	0.971842**
*libzip25/libzip63*	Iin08374(MYB_related)	Corylin	C20H16O4	4	-0.96235**
*libzip25*	Iin10919(NF-YA)	Corylin	C20H16O4	4	0.974904**
*libzip25/libzip63*	Iin21617(ERF)	Corylin	C20H16O4	4	0.988230**
*libzip25*	Iin22265(MIKC_MADS)	Corylin	C20H16O4	4	0.986034**
*libzip25*	Iin10919(NF-YA)	3’-Methoxy Puerarin	C22H22O10	3	0.964215**
*libzip25/libzip63*	Iin21617(ERF)	3’-Methoxy Puerarin	C22H22O10	3	0.954844**
*libzip25*	Iin22265(MIKC_MADS)	3’-Methoxy Puerarin	C22H22O10	3	0.957413**

Correlation>0.9: Positive regulation; Correlation<-0.9: Negative regulation. ** means p-value < 0.01.

The metabolites directly associated with *IibZIP* genes or associated with the regulatory network constructed from the *bZIP* genes collectively included a large number of primary metabolites, flavonoids, phytohormones, and alkaloids, which may reduce plant damage under salt stress. These results indicated that *IibZIP* genes had a synergistic effect with transcription factors to regulate metabolite synthesis, and had certain positive and negative regulatory effects to help the plant to tolerate external stress.

## Discussion

The *bZIP* gene superfamily encodes transcription factors and is widely distributed in eukaryotes. The *bZIP* genes play important roles in plant growth, seed development, secondary metabolism, and stress response (Yanyan et al., 2018). The dried leaves and roots of *I. indigotica* are a traditional Chinese medicine used to treat diseases and viruses, and are used as raw materials in the dye industry, hence the species is an important medicinal and industrial plant cultivated worldwide (Shu-Jen et al., 2012). The *IibZIP* gene family has not previously been identified and characterized in *I. indigotica*, and thus the functions of the *IibZIP* genes were unclear. In this study, 65 *bZIP* family members were identified in *I. indigotica* and classified into 11 subgroups after removal of incomplete domains and redundant sequences. The number and subgroup classification of the *IibZIP* genes were similar to those of other plants. For example, *A. thaliana* has 75 *AtbZIP* genes, *Pyrus bretschneideri* has 62 *PbbZIP* genes (Aamir et al., 2021), *Cannabis sativa* has 51 *CsbZIP* genes (Meng et al., 2022), and *Solanum tuberosum* has 49 *StbZIP* genes (Qi et al., 2021), all of which are classified into 11 subgroups. Further analysis revealed the presence of four *bZIP* genes in the H subgroup (comprising *HY5* transcription factors) of *I. indigotica*, which was higher than the number known in *A. thaliana* (two), *C. sativa* (one), *S. tuberosum* (one), and *Fagopyrum tataricum* (two) (Liu et al., 2019). The three *HY5* transcription factors (*IibZIPI4*, *IibZIP16*, and *IibZIP47*) and one *HYH* transcription factor (*IibZIPI35*) in the H subgroup of *I. indigotica* suggested that the *IibZIP* gene family may have undergone significant gene expansion during the evolutionary process, and these expanded genes may play an important role in the growth and development of *I. indigotica*.

The analysis of gene structure, conserved domains, and motifs provides the basis for the classification of gene families (Xu et al., 2020). Combinatorial analysis of the *IibZIP* gene structure and phylogenetic tree showed that the coding sequence and untranslated regions, exons and introns, conserved domains and motifs were closely associated with the taxonomy in quantity and distribution. For example, members of subgroup B only have motif 1 and one intron; F subgroup members all have motifs 1 and 10, and the number of introns is 1–3. These findings are consistent with the results previously reported for most *bZIP* gene families (Li et al., 2016). Interestingly, in addition to the H subgroup members having HY5-like domains, the same domains were detected in two members of subgroup B (*IibZIP41* and *IibZIP62*) and one member of subgroup K (*IibZIP26*), which has not been reported previously in other plant species.

Abiotic stress is an important limiting environmental factor for plant growth and development, which can seriously impair plant productivity. When exposed to stress, transcription factors regulate the expression levels of downstream target genes to reduce plant stress-related damage. Therefore, identification of transcription factors involved in stress tolerance is considered to be a powerful strategy to improve crop stress tolerance (Tomas et al., 2020). The ability of *bZIP* transcription factors to enhance plant tolerance to abiotic stress has been extensively reported; for example, in chrysanthemum, overexpression of *DgbZIP3* and *DgbZIP2* increases cold tolerance. Interestingly, *DgbZIP3* and *DgbZIP2* can also interact to regulate the expression of *DgPOD*, which can improve the tolerance of chrysanthemum to cold stress by promoting the increase of peroxidase activity and regulating the balance of reactive oxygen species (Bai et al., 2022). Overexpression of *GmbZIP2* in *A.thaliana* and soybean hairy roots can improve plant tolerance to salt and drought stress, and significantly increased expression of the soybean stress-responsive genes *GmDHN15*, *GmMYB48*, *GmLEA*, and *GmWD40* is detected in transgenic hairy roots (Yang et al., 2020). Blueberry inoculation with dark septate endophytes (DSEs) can increase the expression of *VabZIP12* to improve tolerance to salt stress, and overexpression of *VabZIP12* in *A.thaliana* increases the activity of enzymatic antioxidants and up-regulates related genes to enhance salt stress tolerance (Dehui et al., 2022). The bZIP transcription factor HY5 binds to the G-box and C-box elements of *Δ1-PYRROLINE-5-CARBOXYLATE SYNTHASE 1* (*P5CS1*) and the C-box element of *PROLINE DEHYDROGENASE 1* (*PDH1*) to promote proline biosynthesis and help *A.thaliana* cope with salt and drought stress (Kovács et al., 2019). In the present study, 14 differentially expressed *IibZIP* genes were detected in leaves of *I. indigotica* under salt stress, of which the expression levels of nine genes (*IibZIP4*, *IibZIP23*, *IibZIP28*, *IibZIP34*, *IibZIP37*, *IibZIP44*, *IibZIP51*, *IibZIP53*, and *IibZIP56*) were significantly increased under both 24 h and 48 h salt stress. The gene *IibZIP4* is a homolog of *AtHY5*, and thus may perform the same anti-stress function as *HY5.* The genes *IibZIP28*, *IibZIP37*, and *IibZIP56* have *cis*-acting elements involved in defense and stress responsiveness and in low-temperature responsiveness. The genes *IibZIP23*, *IibZIP34*, and *IibZIP51* formed co-expression associations with MYB, bHLH, and other stress tolerance-related transcription factor family members. It is speculated that these genes may play an important role in helping *I. indigotica* to tolerate salt stress.

Secondary metabolites are a class of organic small molecular compounds produced during the secondary metabolism of plants. The accumulation of secondary metabolites plays an important role in the quality assessment of food crops and Chinese medicinal materials, and secondary metabolites also play a crucial role when plants are under stress (Adinath et al., 2022). Many previous reports have shown that *bZIP* genes function in regulating the synthesis of plant secondary metabolites, including alkaloids, flavonoids, and other substances. The abscisic acid-induced apple *bZIP* transcription factor *MdbZIP44* can interact with *MdMYB1* to enhance the binding to downstream target gene promoters to promote the anthocyanin response to ABA, thereby promoting anthocyanin synthesis (Jian-Ping et al., 2018). *SlHY5* directly recognizes and binds to the G-box and ACGT elements in the promoters of the anthocyanin biosynthesis genes dihydroflavonol 4-reductase (*DFR*), chalcone synthase 1 (*CHS1*), chalcone synthase 2 (*CHS2*), and thereby enhance anthocyanin accumulation in tomato (Liu et al., 2018). The biosynthesis of medicinally valuable terpenoid indole alkaloids (TIAs) in periwinkle is regulated by the transcriptional activator *CrMYC2*, and overexpression of *CrMYC2* can enhance the expression of the *bZIP* transcription factor *CrGBF* and reduce the accumulation of alkaloids in the hairy roots of periwinkle. Furthermore, *CrGBF1* and *CrGBF2* form homo- and heterodimers to repress the transcriptional activity of key TIA pathway gene promoters to reduce TIA biosynthesis (Sui et al., 2018). Similarly, *bZIP* genes regulate the biosynthesis of primary metabolites in plants; for example, overexpression of *SlbZIP1* in tomato and overexpression of *AtbZIP11* in A.haliana significantly up-regulates ASPARAGINE SYNTHETASE 1 (*ASN1*) and expression of PROLINE DEHYDROGENASE 2 (*ProDH2*), and influences amino acid content content (Dietrich et al., 2011). In the present study, by integrating transcriptomic and metabolomic data for *I. indigotica* leaves under salt stress and performing an association analysis, we determined that seven *IibZIP* genes were directly positively correlated with 32 compounds. Extracting and analyzing the genes and compounds with the highest correlation coefficients, the genes *IibZIP23*, *IibZIP38*, and *IibZIP51* were significantly positively correlated with six compounds, comprising the alkaloids stylopine, tabersonine, and indole-3-acetic acid, the flavonoid myricetin 3-O-galactoside, and the primary metabolites 2-hydroxy-6-aminopurine and 3-dehydroshikimic acid. Taking the *bZIP* genes as the center of the regulatory network, 10 *IibZIP* genes, 24 transcription factors, and 44 metabolites were strongly associated. Extracting and analyzing the genes and compounds with the strongest correlations, the *IibZIP* genes *IibZIP23* and *IibZIP63*, and MYB and ERF transcription factor family members were significantly correlated with four compounds, comprising the flavonoids corylin and 3′-methoxy puerarin, the indole alkaloid abrine, and the primary metabolite xanthosine dihydrate. Their correlations included both positive and negative correlations. These related metabolite species are consistent with the functions of a large number of reported *bZIP* genes in regulating metabolite synthesis. It is speculated that these *bZIP* genes may regulate the synthesis of different kinds of metabolites to tolerate external stress in *I. indigotica*. Their functions in *I. indigotica* remain to be verified by further experiments.

## Conclusion

This study used multi-omics technology to identify and functionally mine the *bZIP* gene family of *I. indigotica*. A total of 65 *IibZIP* genes were identified in the *I. indigotica* genome and their chromosomal location, structural features, evolutionary relationships, and expression patterns were analyzed. Correlation analysis was conducted between transcriptomic and metabolomic data for *I. indigotica* leaves exposed to salt stress. The *IibZIP* genes that potentially regulated the synthesis of alkaloids, flavonoids, and primary metabolites were mined. The present results provide a basis for further functional analysis of *IibZIP* genes to elucidate the regulatory network by which *IibZIP* transcription factors control metabolite synthesis under salt stress, and provide a reference for taxonomic and functional studies of the *bZIP* gene family in other plant species.

## Data availability statement

The original contributions presented in the study are publicly available. This data can be found here: NCBI, PRJNA854335. Publicly available datasets were analyzed in this study. This data can be found here: https://doi.org/10.6084/m9.figshare.8940377.v1 https://doi.org/10.6084/m9.figshare.8940383.v1.

## Author contributions

All authors contributed to the study conception and design. Material preparation, and data collection and analysis were performed by MJ and ZW. RC, SY, PZ, and NX designed the experiment and performed data analysis. The manuscript was drafted by MJ, WZ, HL, and WM. All authors commented on previous versions of the manuscript. All authors read and approved the final manuscript.

## Funding

Qiqihar Academy of Medical Sciences project (QMSI2021M-13); The youth team construction project of the central government’s special support for the development and reform of local colleges and Universities (21B023); National key research and development projects, key technologies common to rural industries, collection and screening of ginseng and other authentic medicinal materials, and research and demonstration of breeding technology (2021YFD1600901). Heilongjiang Touyan Innovation Team Program (Grant Number: [2019] No. 5)

## Conflict of interest

The authors declare that the research was conducted in the absence of any commercial or financial relationships that could be construed as a potential conflict of interest.

## Publisher’s note

All claims expressed in this article are solely those of the authors and do not necessarily represent those of their affiliated organizations, or those of the publisher, the editors and the reviewers. Any product that may be evaluated in this article, or claim that may be made by its manufacturer, is not guaranteed or endorsed by the publisher.
